# Postmeal triglyceridemia and variability of HbA1c and postmeal glycemia were predictors of annual decline in estimated glomerular filtration rate in type 2 diabetic patients with different stages of nephropathy

**DOI:** 10.1186/s40200-016-0284-0

**Published:** 2017-01-11

**Authors:** Ayaka Tsuboi, Akiko Takenouchi, Miki Kurata, Keisuke Fukuo, Tsutomu Kazumi

**Affiliations:** 1 Research Institute for Nutrition Sciences, Mukogawa Women’s University, 6-46, Ikebiraki-cho, Nishinomiya, Hyogo 663-8558 Japan; 2 Department of Nutrition, Osaka City Juso Hospital, Osaka, 532-0034 Japan; 3 Department of Food Sciences and Nutrition, School of Human Environmental Sciences, Mukogawa Women’s University, 6-46, Ikebiraki-cho, Nishinomiya, Hyogo 663-8558 Japan; 4 Diabetes Division, Kohnan Kakogawa Hospital, Kakogawa, Hyogo, 675-0005 Japan

**Keywords:** Postmeal glucose, HbA1c, Coefficient of variation, Postmeal triglycerides, Kidney function, eGFR

## Abstract

**Background:**

This study examined associations of annual glycemic variability and postprandial dysmetabolism with annual decline in estimated glomerular filtration rate (eGFR) in type 2 diabetic patients with different stages of nephropathy.

**Methods:**

Intrapersonal mean and coefficient of variation (CV) of HbA1c, fasting and postmeal concentrations of plasma glucose (FPG and PMPG, respectively) and serum triglycerides (FTG and PMTG, respectively) during the first 12 months after enrollment were calculated in a cohort of 168 type 2 diabetic patients: 53 with optimal albumin/creatinine ratio (ACR < 10 mg/g), 62 with high normal ACR (10–29 mg/g) and 53 with elevated ACR (≧30 mg/g). Annual changes in eGFR were computed using 52 (median) creatinine measurements obtained over a median follow-up of 6.0 years. Multivariate linear regressions assessed the independent correlates of changes in eGFR.

**Results:**

Kidney function declined faster in patients with high normal and elevated ACR (−1.47 and −2.01 ml/min/1.73 m^2^/year, respectively) compared to patients with optimal ACR (0.08 ml/min/1.73 m^2^/year, *p* < 0.05). In patients with high normal ACR, age (standardized β、-0.30、*p* = 0.01), CV-HbA1c (standardized β、-0.66、*p* < 0.001) and CV-PMPG (standardized β、-0.27、*p* = 0.01) was associated with annual eGFR decline independently of mean HbA1c and PMPG, sex, BMI, waist circumference, diabetes duration and therapy, means and CVs of FPG and systolic blood pressure, baseline eGFR, log ACR and uses of anti-hypertensive medications (R^2^ = 0.47). In patients with elevated ACR, PMTG (standardized β、-0.408, *p* = 0.007) was associated with annual eGFR decline (R^2^ = 0.15).

**Conclusions:**

Consistency of glycemic control and management of postprandial glycemia and lipidemia are important to preserve kidney function in type 2 diabetic patients.

## Background

The incidence and prevalence of chronic kidney disease are increasing [1], and it has been recognized as a medical, social, and economic problem worldwide. A mild decline of kidney function increases the risk of cardiovascular disease in the general population [2]. Therefore, the early detection and aggressive modification of risk factors for the decline of kidney function are important. Aging, hypertension, and diabetes mellitus are the most common risk factors for the development of chronic kidney disease [3, 4]. In addition to hypertension and diabetes, dyslipidemia has an important role in the progression of kidney disease in patients with diabetes [5]. Longitudinal studies have found an association between fasting serum triglycerides (FTG) and the development of chronic renal insufficiency [6–9]. However, we are not aware of report that examined associations of postmeal TG (PMTG) with changes in kidney function despite the fact that the vasculature is commonly exposed to prolonged and exaggerated postprandial triglyceridemia, especially in type 2 diabetic patients [10].

There is emerging interest to examine the influence of glycemic and BP variance in diabetic vascular complications [11, 12]. Recently, we have shown direct association of HbA1c variability and albuminuria with kidney function decline in type 2 diabetic patients [13]. We, therefore, asked the question whether glycemic variability, fasting and postmeal TG might directly associated with annual decline in estimated glomerular filtration rate (eGFR) in patients with type 2 diabetes with different stages of nephropathy.

## Methods

The setting for this observational study was the same as previously reported [13]. Study protocol was consistent with the Japanese Government’s Ethical Guidelines Regarding Epidemiological Studies in accordance with the Declaration of Helsinki. Patients with hepatitis B surface antigen or antibodies against hepatitis C virus were excluded. Those who had aspartate aminotransferase and alanine aminotransferase of 100 U/L or greater, serum creatinine≧2.0 mg/dl were excluded as well. We examined a cohort of 168 patients with type 2 diabetes in whom 153 patients (91%) had 12 monthly visits with blood samplings [13]. They had been regularly attending the clinic in 2004 and 2005. They were enrolled in the study at the first visit in 2005 and followed up for the subsequent at least 24 months through December 31, 2012 to assess kidney function with a median follow-up of 6.0 years (interquartile range; 4.1–6.5 years). In the 153 patients, blood was withdrawn on 2 occasions; at 2 h after breakfast taken at home and after an overnight fasting. This was done every other month. In the remaining 15 patients, blood was obtained after an overnight fasting.

For each subject on each monthly visit, waist circumference, weight and BP were measured by registered nurses. Plasma glucose (PG) was determined by the glucose oxidase method using an autoanalyzer (Glucoroder MAX, A&T, Yokohama, Japan). Serum lipids and lipoproteins, creatinine, hepatic enzymes, uric acid and other blood tests were measured by standard methods using an autoanalyzer (H7600, Hitachi, Tokyo, Japan). HbA1C values were determined by high performance liquid chromatography (HLC723-G7, Tosoh, Tokyo, Japan). Urinary albumin was measured once during the first 3–4 months after enrollment in random urine samples using a turbidimetric immunoassay and expressed as albumin /creatinine ratio (ACR). Serum and urinary creatinine were measured enzymatically and estimated glomerular filtration rate (eGFR) was determined using the equation recommended by the Japanese Society for Nephrology [14].

Microalbuminuria was defined as ACR between 30 and 299 mg/g [15]. The recommended normal range was further subdivided into high normal ACR (10–29 mg/g) and optimal ACR (<10 mg/g) [16]. Because relatively few participants (*n* = 6) had macroalbuminuria (ACR ≥300 mg/g), these participants were grouped with those who had microalbuminuria and were termed as elevated ACR.

Intrapersonal mean and coefficient of variation (CV) of HbA1c, fasting and postmeal plasma glucose (FPG and PMPG, respectively) and serum triglycerides (FTG and PMTG, respectively) taken during the first 12 months after enrollment were calculated; 153 patients (91%) had 12 measurements of HbA1c and systolic BP, and 6 measurements of FPG, PMPG, FTG and PMTG, respectively. Linear regression was used to estimate changes in eGFR using a median of 52 creatinine measurements (interquartile range; 31–60) over 6.0 years of follow-up in each patient.

Data were presented as mean ± SE unless otherwise stated. Differences between 2 groups were analyzed by *t* test and frequencies of conditions by Chi-square tests. Differences among 3 groups were analyzed using analysis of variance and then Bonferroni’s multiple comparison procedure was done. Correlations of annual eGFR decline were evaluated by Pearson correlation analysis. Multiple linear regression analyses were performed to further identify the most significant variables contributing to annual eGFR decline. Potential confounders were forced into the model and standardized β coefficients were calculated. A two-tailed *P* < 0.05 was considered statistically significant. All calculations were performed with SPSS system 15.0 (SPSS Inc., Chicago, IL).

## Results

As previously reported [13], patients had relatively good glycemic, lipid and BP control. Baseline means of eGFR were means of 2–4 measurements during the first 3–4 months after enrollment, and averaged 76 ± 16 ml/min/1.73 m^2^. Changes in eGFR were linear and averaged −1.05 ± 3.39 ml/min/1.73 m^2^ per year. At baseline, 27 (16.0%) of 168 patients had eGFR < 60 ml/min/1.73 m^2^ and 53, 62 and 53 patients had optimal, high normal and elevated ACR (microalbuminuria 47, macroalbuminuria 6), respectively.

Patients with elevated compared to optimal ACR were older, had higher mean HbA1c, FPG and PMPG (Table 1). Patients with elevated ACR had higher FTG and systolic BP and lower HDL cholesterol. In patients with high normal and elevated ACR eGFR decreased whereas eGFR did not change in patients with optimal ACR. There was no difference in baseline eGFR among 3 groups and other variables.

**Table 1:** Baseline features of type 2 diabetic patients with optimal, high normal and elevated urinary albumin/creatinine ratio (ACR)

	Optimal	High normal	Elevated
	ACR <10 mg/g	ACR: 10–29 mg/g	ACR >30 mg/g
	(*n* = 53)	(*n* = 62)	(*n* = 53)
Male sex (n, %)	32,61.5	26,42.6	31,58.5
Smokers (n, %)	15,28.8	22,36.1	20,37.7
Age (years)	59.4 ± 1.4^a^	63.0 ± 1.2^a,b^	64.2 ± 1.4^b^
BMI (kg/m^2^)	23.9 ± 0.4	23.9 ± 0.4	25.0 ± 0.6
Waist circumference (cm)	85.3 ± 1.1	86.6 ± 1.0	89.1 ± 1.9
Duration of diabetes (years)	9.3 ± 0.9	10.1 ± 1.0	10.1 ± 1.1
Treatment of			
Diabetes;diet/OHA/insulin (%)	44/42/14	25/57/18	26/53/21
Hypertension; CCB/RASi/diuretics (%)	33/39/6	26/34/3	47/53/6
HbA1c (%)	6.8 ± 0.1^a^	7.0 ± 0.1^a,b^	7.3 ± 0.1^b^
Fasting PG (mg/dl)	120 ± 3^a^	125 ± 3^a,b^	130 ± 3^b^
Post-breakfast PG (mg/dl)	137 ± 6^a^	155 ± 6^b^	169 ± 8^b^
CV-HbA1c (%)	6.5 ± 1.1	6.5 ± 0.7	8.0 ± 0.8
CV-Fasting PG (%)	12.0 ± 1.0	15.4 ± 1.3	14.4 ± 1.2
CV-Post-breakfast PG (%)	19.7 ± 1.2	22.9 ± 1.6	23.0 ± 1.9
Total cholesterol (mg/dl)	191 ± 3	188 ± 3	186 ± 3
LDL cholesterol (mg/dl)	113 ± 2	111 ± 3	110 ± 4
HDL cholesterol (mg/dl)	59 ± 2^a^	55 ± 2^a,b^	53 ± 2^b^
Fasting TG (mg/dl)	102 ± 6^a^	112 ± 5^a,b^	130 ± 9^b^
Post-breakfast TG (mg/dl)	134 ± 9	146 ± 8	159 ± 10
Serum creatinine (mg/dl)	0.77 ± 0.02	0.72 ± 0.02	0.78 ± 0.03
eGFR (ml/min/1.73 m^2^)	75.1 ± 1.8	77.0 ± 2.0	75.3 ± 2.7
⊿eGFR (ml/min/1.73 m^2^/year)	0.08 ± 0.33^a^	−1.47 ± 0.43^b^	−2.01 ± 0.40^b^
Uric acid (mg/dl)	5.23 ± 0.16	4.92 ± 0.17	5.50 ± 0.20
Systolic BP (mmHg)	126 ± 2^a^	127 ± 1^a^	132 ± 2^b^
CV-Systolic BP (%)	7.7 ± 0.3	8.1 ± 0.3	8.3 ± 0.3
Diastolic BP (mmHg)	73 ± 1	72 ± 1	72 ± 1
Urinary ACR (mg/g)	6.4 ± 0.4^a^	17.2 ± 0.6^a^	236 ± 75^b^
log ACR	0.7 ± 0.0^a^	1.2 ± 0.0^b^	1.9 ± 0.1^c^

Mean ± SE or %. *OHA* oral hypoglycemic agents, *CCB* calcium channel blockers, *RASi* renin-angiotensin system inhibitors, *PG* plasma glucose, *CV* coefficient of variation, *eGFR* estimated glomerular filtration rate, ⊿eGFR annual changes in eGFR, *BP* blood pressure, *ACR* albumin/creatinine ratio

Means not sharing common alphabetical letters are significantly different each other at *p* < 0.05 or less. Means without a letter indicate no statistical significance among 3 groups.

**Fig. 1:**
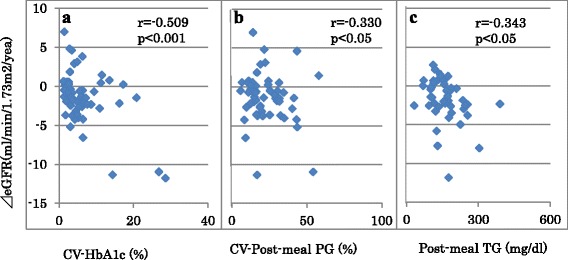
Scatter plots between annual changes in estimated glomerular filtration rate (⊿eGFR) and coefficient of variation (CV) of HbA1c **a** and post-meal plasma glucose (PG, **b**) in diabetes patients with high normal albuminuria, and post-meal triglyceride (TG) in diabetes patients with elevated albuminuria **c**. High normal and elevated albuminuria: urinary albumin/creatinine ratio between 10 and 29 mg/g and ≧30 mg/g, respectively

In patients with high normal ACR, annual eGFR change was inversely associated with CV-HbA1c, CV-FPG and CV-PMPG but not with means of the 3 glycemic variables (Table 2). However, annual eGFR decline did not show significant associations with age, sex, duration of diabetes, baseline eGFR, treatment for diabetes, lipids and BP variables. Multiple linear regression analysis revealed that age (standardized β、-0.30、*p* = 0.01), CV-HbA1c (standardized β、-0.66、*p* < 0.001) (Fig. 1a) and CV-PMPG (standardized β、-0.27、*p* = 0.01) (Fig. 1b) was associated with annual eGFR decline independently of mean HbA1c and PMPG, sex, BMI, waist circumference, diabetes duration and therapy, means and CVs of FPG and systolic blood pressure, baseline eGFR, log ACR and uses of anti-hypertensive medications (R^2^ = 0.47).

**Table 2:** Correlation coefficients of annual changes in estimated glomerular filtration rates in type 2 diabetic patients with optimal, high normal and elevated urinary albumin/creatinine ratio (ACR)

ACR status	Optimal (ACR < 10)	High normal (ACR;10–29)	Elevated (ACR≧30)
Male sex (n, %)	−0.163	−0.008	0.207
Smokers (n, %)	−0.182	−0.226	−0.022
Age (years)	−0.115	−0.028	0.175
BMI (kg/m^2^)	−0.232	0.094	−0.110
Waist circumference (cm)	−0.277	−0.030	−0.099
Duration of diabetes (years)	−0.242	−0.170	0.081
Treatment of			
diabetes;diet/OHA/insulin (%)	−0.133	−0.173	−0.140
hypertension;CCB/RASi/diuretics (%)	−0.286	−0.134	0.292*
HbA1c (%)	−0.094	−0.097	0.045
Fasting PG (mg/dl)	−0.096	−0.148	0.113
Post-breakfast PG (mg/dl)	−0.083	−0.080	0.086
CV-HbA1c (%)	0.164	−0.509***	−0.400**
CV-Fasting PG (%)	−0.050	−0.424**	−0.233
CV-Post-breakfast PG (%)	−0.067	−0.330*	−0.056
Total cholesterol (mg/dl)	0.103	−0.067	0.114
LDL cholesterol (mg/dl)	0.000	−0.094	0.083
HDL cholesterol (mg/dl)	0.130	−0.009	0.170
Fasting TG (mg/dl)	−0.091	0.013	−0.257
Post-breakfast TG (mg/dl)	−0.054	0.014	−0.343*
Serum creatinine (mg/dl)	0.029	0.006	−0.132
eGFR (ml/min/1.73 m^2^)	0.013	−0.128	−0.034
⊿eGFR (ml/min/1.73 m^2^/year)	1.000	1.000	1.000
Uric acid (mg/dl)	−0.040	−0.082	−0.080
Systolic BP (mmHg)	−0.164	−0.109	0.302*
CV-Systolic BP (%)	−0.107	−0.156	0.064
Diastolic BP (mmHg)	−0.085	−0.002	0.146
Urinary ACR (mg/g)	−0.012	−0.157	−0.281*
log ACR	−0.077	−0.169	−0.034

Abbreviations are the same as in Table 1 *: *p* < 0.05, **: *p* < 0.01, ***: *p* < 0.001

In patients with elevated ACR, annual eGFR decline was inversely associated with CV-HbA1c, PMTG and urinary ACR and positively with treatment of hypertension and systolic BP (Table 2). However, annual eGFR decline did not show significant association with any other variables. Multiple linear regression analysis revealed that PMTG (standardized β、-0.408, *p* = 0.007) (Fig. 1c) was associated with annual eGFR decline independently of CV-HbA1c, age, sex, BMI, waist circumference, duration and treatment of diabetes, means and CVs of HbA1c, FPG and PMPG, FTG, baseline eGFR, log ACR and uses of anti-hypertensive and lipid-lowering medications (R^2^ = 0.15).

There was no significant association between eGFR changes and any variables studied in patients with optimal ACR (Table 2).

## Discussion

The current study is the first to demonstrate direct associations of annual variation in HbA1c and PMPG with annual decline in eGFR in patients with type 2 diabetes and high normal ACR. In addition, the current study is also the first to demonstrate direct associations of PMTG with annual decline in kidney function in type 2 diabetic patients with elevated ACR. These associations were independent of mean HbA1c and PMPG, FTG and known predictors of GFR decline [17]. The current finding that kidney function decline was faster in patients with high normal compared to optimal ACR has confirmed previous studies that urinary albumin, even in the microalbuminuric range, is a predictor of renal function impairment [17–20].

As mentioned earlier, longitudinal studies have found an association between FTG and renal replacement therapy in type 2 diabetic patients [6], GFR decline in patients with chronic glomerulonephritis [7], a rise in serum creatinine of 0.4 mg/dL or greater in the general population [8] and development of renal impairment in type2 diabetic patients [9]. In the UK prospective Diabetes Study [21], high levels of FTG were a independent risk factor of micro- and macro-albuminuria. The present study is the first to demonstrate that PMTG but not FTG was associated with annual GFR decline in type 2 diabetic patients with nephropathy (elevated ACR). CV of HbA1c, which showed strong association in univariate analysis, did not emerge as an independent predictor of annual eGFR decline in patients with elevated ACR, although it was independently associated with annual eGFR decline in the entire 168 patients as recently reported [13].

Recent meta-analyses [11, 22] indicated that higher HbA1C variability was independently associated with higher risk of renal disease in both type 1 and 2 diabetes patients. Our previous study was the first to show a direct association between annual HbA1c variability and kidney function decline and demonstrated stronger association in type 2 diabetic patients with elevated ACR than in patients with normoalbuminuria [13]. In the present study, patients with normoalbuminuria were divided into 2 groups: those with optimal and high normal ACR and 2 groups were analyzed separately. Annual eGFR decline was independently associated with CV of HbA1c in patients with high normal ACR but not in patients with optimal and elevated ACR.

In a recent systematic review [23], only 2 studies assessed the association of postprandial glucose with retinopathy [24, 25] but no report with diabetic kidney disease. The current study is the first to demonstrate direct associations of annual variation in PMPG but not mean PMPG with annual decline in eGFR in type 2 diabetic patients with high normal ACR. We are aware of only 1 prospective study [26] assessing the relationship between variation in FPG and risk of kidney disease in type 2 diabetic patients. Lin et al. [26] reported that annual variation in HbA1c and FPG was independently associated with risk of diabetic nephropathy (eGFR < 60 mL/min/1.73 m^2^). Among 4399 patients with type 2 diabetes in the intensive group of the ADVANCE trial [27], SD of FPG and HbA1c independently predicted future combined microvascular events (nephropathy or retinopathy).

Several mechanisms including oxidative stress and inflammation may be involved in the association between glycemic variability and outcomes as recently discussed in detail [11]. Current findings of independent associations of PMTG and CV-PMPG with annual decline in kidney function in type 2 diabetic patients may be in line with the observation of an independent effect of postprandial hypertriglyceridemia and hyperglycemia on endothelial dysfunction and oxidative stress generation in both diabetic and normal subjects [28].

Type 2 diabetic patients with high normal and elevated ACR in the present study had annual eGFR decline (−1.47 and −2.01 ml/min/1.73 m^2^ per year, respectively) which was comparable to non-diabetic Japanese patients with early-stage chronic kidney disease (eGFR > 60 ml/min/1.73 m^2^) (−1.64 ml/min/1.73 m^2^ per year) [29]. Further, annual eGFR decline of our patients was slower than the rate found in Japanese type 2 diabetic patients without clinical albuminuria (−2.94 ml/min/1.73 m^2^ per year) [30] despite comparable baseline eGFR. These findings may be due in part to the fact that our patients had better glycemic (mean HbA1c; 7.0–7.3 vs. 8.4%) and BP (127–132 vs. 135 mmHg) control. Slower eGFR decline associated with better diabetic control in our patients may be related to failure to detect association between mean HbA1c and annual eGFR decline in the present study.

The strength of the current study is that we used 12-month period when mean HbA1c and HbA1c variability were calculated from 12 measurements in 91% participants. Postmeal TG were measured after breakfast eaten at home: in real-life conditions. In addition, serum creatinine and hence eGFR during follow-up period were measured much more frequently than in previous studies as mentioned elsewhere [13]. This could contribute to the reliability of changes in kidney function. Such a testing frequency is routine in clinical settings in Japan. However, frequent measures of HbA1c may artificially inflate precision and decrease standard deviation, which may impact the results. Finally, BP control and variability and postprandial TG also have been taken into accounted. Major limitations are that study participants were small in number and from a single clinic in Japan. However, the characteristics of our study participants are similar to those reported in a previous large-scale study in Japan [31]. Another limitation was that urinary ACR was measured once in a random urine sample. Since the albumin excretion variability is too high, classification of patients according to single measurement of ACR may be unreliable.

## Conclusions

The current study has demonstrated that predictors of annual decline in kidney function differed in type 2 diabetic patients with different stages of nephropathy. These findings suggest that more attention should be paid by clinicians in diabetes control, avoiding excessive oscillations in blood glucose levels in type 2 diabetic patients with high normal ACR. In patients with ACR≧30 mg/g, management of postmeal TG may be important because normotriglyceridemic patients with type 2 diabetes and microalbuminuria have an almost 3-fold higher postprandial triglyceridemia than patients without microalbuminuria after ingestion of a mixed test meal [32]. Further studies are needed to confirm the association in other ethnic groups with more patients.

## Abbreviations

ACR: Albumin /creatinine ratio
BP: Blood pressure
CV: Coefficient of variation
eGFR: estimated glomerular filtration rate
FPG: Fasting plasma glucose
FTG: Fasting serum triglycerides
PMPG: Post-meal plasma glucose
PMTG: Post-meal serum triglycerides.
